# Theory of Robot Mind: False Belief Attribution to Social Robots in Children With and Without Autism

**DOI:** 10.3389/fpsyg.2019.01732

**Published:** 2019-08-09

**Authors:** Yaoxin Zhang, Wenxu Song, Zhenlin Tan, Yuyin Wang, Cheuk Man Lam, Sio Pan Hoi, Qianhan Xiong, Jiajia Chen, Li Yi

**Affiliations:** ^1^School of Psychological and Cognitive Sciences, Beijing Key Laboratory of Behavior and Mental Health, Peking University, Beijing, China; ^2^South China Academy of Advanced Optoelectronics, South China Normal University, Guangzhou, China; ^3^Department of Psychology, Sun Yat-sen University, Guangzhou, China; ^4^Institute of Psychology, Chinese Academy of Sciences, Beijing, China; ^5^Department of Electrical Engineering, Chalmers University of Technology, Gothenburg, Sweden

**Keywords:** autism spectrum disorder, social robot, false belief, children, theory of mind

## Abstract

This study aims to probe how children with and without autism spectrum disorders (ASD) attribute false belief to a social robot and predict its action accordingly. Twenty 5- to 7-year-old children with ASD and 20 age- and IQ-matched typically developing (TD) children participated in two false belief tasks adapted for robot settings (change-of-location task and the unexpected-contents task). The results showed that most TD children are capable of attributing false belief to the social robot, that is, they could infer higher level mental states in robots, which extends our understanding in TD children’s perception and cognition on social robots. Conversely, children with ASD still show difficulty in interpreting robots’ mental states compared to their TD peers, which would greatly interfere with their interactions and communications with social robots and might impact on efficiency of robot-based intervention and education approaches. This group difference in attributing false belief to social robots could not be explained by the different perception and categorization of the robot. Our study implies that although children with ASD appear to be highly attracted by social robots, they still have difficulty in understanding mental states when socially interacting with robots, which should be taken into consideration when designing the robot-based intervention approach targeting to improve social behaviors of ASD.

## Introduction

Robots are one of the scientific and technological advances that greatly contribute to the momentous development in our contemporary lives. Robots, especially social robots, have been used in the field of education since they could function as educationists and caregivers with well-designed motor and communication capacities (Fong et al., 2003; Jipson and Gelman, 2007). Over the last decade, people have speculated a promising future for robots and accordingly have applied them in academic and commercial fields. For instance, researchers found that the social robots could serve as knowledgeable interlocutors (Breazeal et al., 2016) and word teachers for children (Moriguchi et al., 2011).

Apart from using robots in typically developing (TD) children, there is also an increasing need for applying the social robot in the interventions for children with special needs, such as children with autism spectrum disorder (ASD). ASD is a neurodevelopmental disorder that is characterized by restricted and repetitive behaviors and social-communication impairments (American Psychiatric Association, 2013). Previous studies have demonstrated that children with ASD showed more interests on the robots compared to non-robotic toys or humans (see Diehl et al., 2012 for a review) and they paid more attention when interacting with robots than humans (Scassellati et al., 2012). Furthermore, using robots to improve their social behaviors is promising. For example, it has been shown that children with ASD improved their joint attention ability when interacting with a robotic system (Warren et al., 2015). Despite the evident advantages of using robots for training and educating children, from a fundamental perspective, it is still unclear how children perceive and understand social robots, which is of key importance for the design and application of robot-based training and education approaches.

This study focused on a fundamental ability in social interaction—Theory of Mind (ToM), which is the ability to infer other people’ mental states (Premack and Woodruff, 1978). Associated with their social communicative impairments, individuals with ASD have been found to be profoundly impaired in ToM-related tasks (e.g., Baron-Cohen, 2001). This ToM hypothesis of autism has been supported by numerous studies using false belief tasks, which measure children’s understanding about others’ false belief (Dennett, 1978; Wellman et al., 2001). If children with ASD display deficits when interacting with the social robot similar as with the human, this would greatly impact on efficiency of robot-based intervention and education approaches.

Meanwhile, some research attention has been paid to examine how TD children attribute mental states to social robots. Kahn et al. (2006, 2012) found that preschoolers could attribute mental states to a robotic pet dog and a humanoid robot, though they disagreed on the proposition that robots possess their own liberty or civil rights. Considering these results, Kahn et al. (2013) proposed that children do not regard humanoid robots as identical to human beings but tend to categorize them into a new ontological entity with peculiarities, referred to as the new ontological category (NOC) hypothesis. In this study, we aimed to examine whether children consider social robots as bearing mental states by focusing on a higher level mental state understanding—the understanding of robots’ false belief. Here, we put a particular focus on how children with ASD would attribute false belief to a social robot when interacting with it.

To this end, we used two conventional false belief tasks: the change-of-location task and unexpected-contents task (Wimmer and Perner, 1983; Gopnik and Astington, 1988; Wimmer and Hartl, 1991). In these tasks, children are told stories in which an agent omits some information and thus holds a false belief about the location or content of an object. Then children are asked to predict the agent’s behavior or mental state about the object as an evaluation of their understanding of the agent’s false belief. Happé (1995) reported that 50% 4-year-old typically developing (TD) children could pass both tasks, while children with ASD showed a significant delay in developing this ability (50% probability of passing at a VMA of 9.17 years). We chose these two false belief tasks since they could easily be modified into interactive tasks, in order to examine how children attribute mental states to robots in an interactive situation. To make the experimental setting similar to intervention situations, we adapted these two conventional false belief tasks by replacing the human agent with a social robot and measured children’s understanding of the robot’s false belief. Considering their impairments in theory of mind, we hypothesized that children with ASD would still manifest deficits in inferring the false belief of the social robot compared to TD group.

## Materials and Methods

### Participants

Twenty Chinese children with ASD (age range 5.08–8.83 years, *M* = 6.79, SD = 0.93; IQ: *M* = 104.90, SD = 11.90; 2 females) and twenty age- and IQ-matched Chinese TD children (age range 5.24–7.32 years, *M* = 6.35, SD = 0.56; IQ: *M* = 106.70, SD = 12.63; 3 females) were recruited. Children with ASD were recruited from communities in two Chinese cities: Beijing and Dongguan. These children with ASD were previously diagnosed by experienced pediatricians from licensed hospitals based on the diagnostic criteria for ASD in the DSM-IV-TR (American Psychiatric Association, 2000). The diagnosis of children with ASD were further confirmed by the Autism Diagnostic Observation Schedule (ADOS; Lord et al., 2000) and Autism Diagnostic Interview-Revised (ADI-R; Lord et al., 1994), or by the Social Responsive Scale (SRS; Constantino and Gruber, 2005) based on parents’ reports. Age- and IQ-matched TD children were recruited and both groups of children’s IQs were assessed using Wechsler Intelligence Scale for Children—Fourth Edition (WISC-IV; Wechsler, 2003). This study was carried out in accordance with the recommendations of the University’s Committee for Protecting Human and Animal Subjects with written informed consent from all subjects’ parents. The protocol was approved by the Committee for Protecting Human and Animal Subjects in School of Psychological and Cognitive Sciences of Peking University, China. All subjects gave written informed consent in accordance with the Declaration of Helsinki.

### Social Robot

We used a social robot *Nao*, produced by Aldebaran Robotics in France. *Nao* is 58-cm high and 5 kg in weight. *Nao* could move flexibly and precisely, listen, speak, and make space-sound positioning. *Nao* attracted children with ASD more than other types of robots (Boucenna et al., 2014). In our study, *Nao* interacted with children by speaking, walking, swinging and so on. We called *Nao* as “*Naonao*,” which sounds like a Chinese child’s name.

### Procedure

Each child, who was tested in a separate room, participated in a warm-up session first. Then, two false belief tasks (the change-of-location task and unexpected-contents task), and a categorization task, were conducted sequentially.

#### Warm-Up Session

The children were asked to participate in a 3-min semi-structured warm-up communication with the social robot. First, the experimenter introduced the robot “*Naonao*” to the children and invited the children to talk with *Naonao* by asking some warm-up questions, such as “what is your name?” or “how old are you?”. The robot answered these questions and then asked similar questions back to the children. This communication familiarized children with the robot and relaxed them when facing the robot.

#### False Belief Tasks

Then the experimenter invited the children to participate in two false belief tasks with the robot, adapted from the traditional change-of-location task (Wimmer and Perner, 1983) and the unexpected-contents task (Perner et al., 1987).

**Figure 1:**
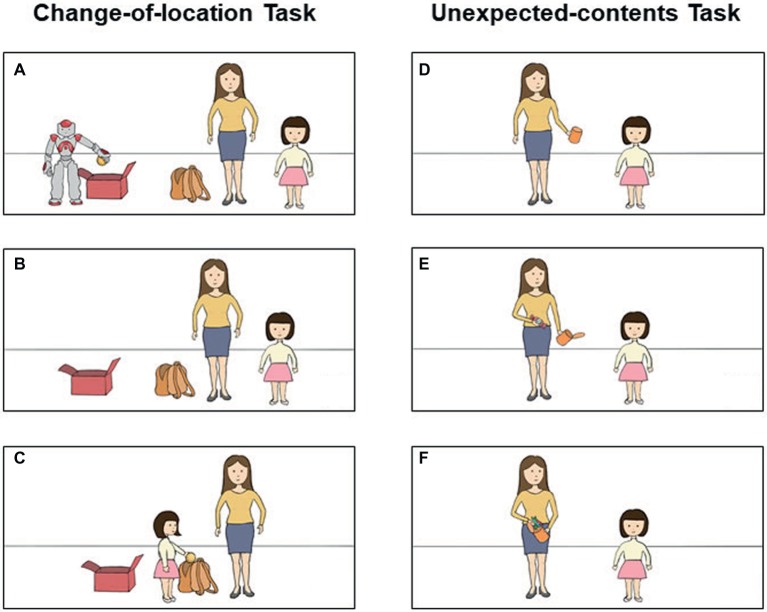
The procedure of change-of-location task **(A–C)** and unexpected-contents task **(D–F)**. In the change-of-location task, children were shown a robot holding a ball saying “this is my ball. I will put it in this box, and come back later to look for it” **(A)**. Then, the robot left the room **(B)** and the experimenter invited children to move the ball from the box and hide into a bag **(C)**. Finally, children were asked the false-belief and two control questions. In the unexpected-contents task, children were shown a candy box and were asked: “What do you think is inside the box?” **(D)**. Following children’s answers, the experimenter opened the box to demonstrate that candies existed there **(E)**. The experimenter then took out all the candies, and replaced them with crayons in front of children **(F)**. Finally, children were asked the false-belief and two control questions.

In the change-of-location task, the children were first shown a performance by the robot: The robot held a ball and said “this is my ball. I will put it in this box and come back later to look for it” (see Figure 1A). Then, the robot left the room (Figure 1B) and the experimenter asked children to move the ball from the box and hide into a bag (Figure 1C). At last, children were asked the following questions: (1) the reality-control question: “Where is the ball now?”, (2) the memory-control question: “Before *Naonao* left the room, where was the ball?” and (3) the false-belief question: “Later, *Naonao* will come back to look for its ball. Where will *Naonao* look for its ball first?” Children’s responses were recorded manually and coded later.

In the unexpected-contents task following the change-of-location task, children were shown a candy box (Figure 1D) and were asked: “What do you think is inside the box?” Regardless of children’s answers, the experimenter opened the box to show that candies existed there (Figure 1E). The experimenter then took out all the candies (Figure 1F), and replaced them with crayons. Then children were asked the following questions: (1) the reality-control question: “What is inside the box now?”, (2) the memory-control question: “What was in the box when you first saw it?” and (3) the false belief question: “*Naonao,* who has never seen this box before, will come back. If I ask *Naonao* what is inside the box, will it^1^ answer the crayons or the candies?”

The reality and memory-control questions in both tasks were designed to confirm that children could understand the task and questions, and that children’s answers to test question (3) reflected their real false belief understanding. The result demonstrated that all children gave correct answers to the reality and the memory-control questions. Thus, we merely counted on the answers to the false-belief questions (3) in our data analyses, and calculated two indices. *Pass rate* was defined as the percentage of children responding correctly to the false-belief question in each task, and the *accuracy* was defined as the percentage of correct answers to the false-belief questions per child. We compared the group differences of pass rates and accuracy.

#### Categorization Task

A categorization task was employed to examine how children would perceive and categorize the robot into four related categorizations: toys, human, animals, and machine. This task was adapted from the research of Peca et al. (2014) by presenting children a card depicting four entities for each category. Children were shown all four cards at the same time and asked, “which of these four cards do you think most resembles *Naonao*?” We examined the group difference in the performance of this categorization task, and the correlation between the performance of the categorization task and the false belief tasks.

## Results

**Figure 2:**
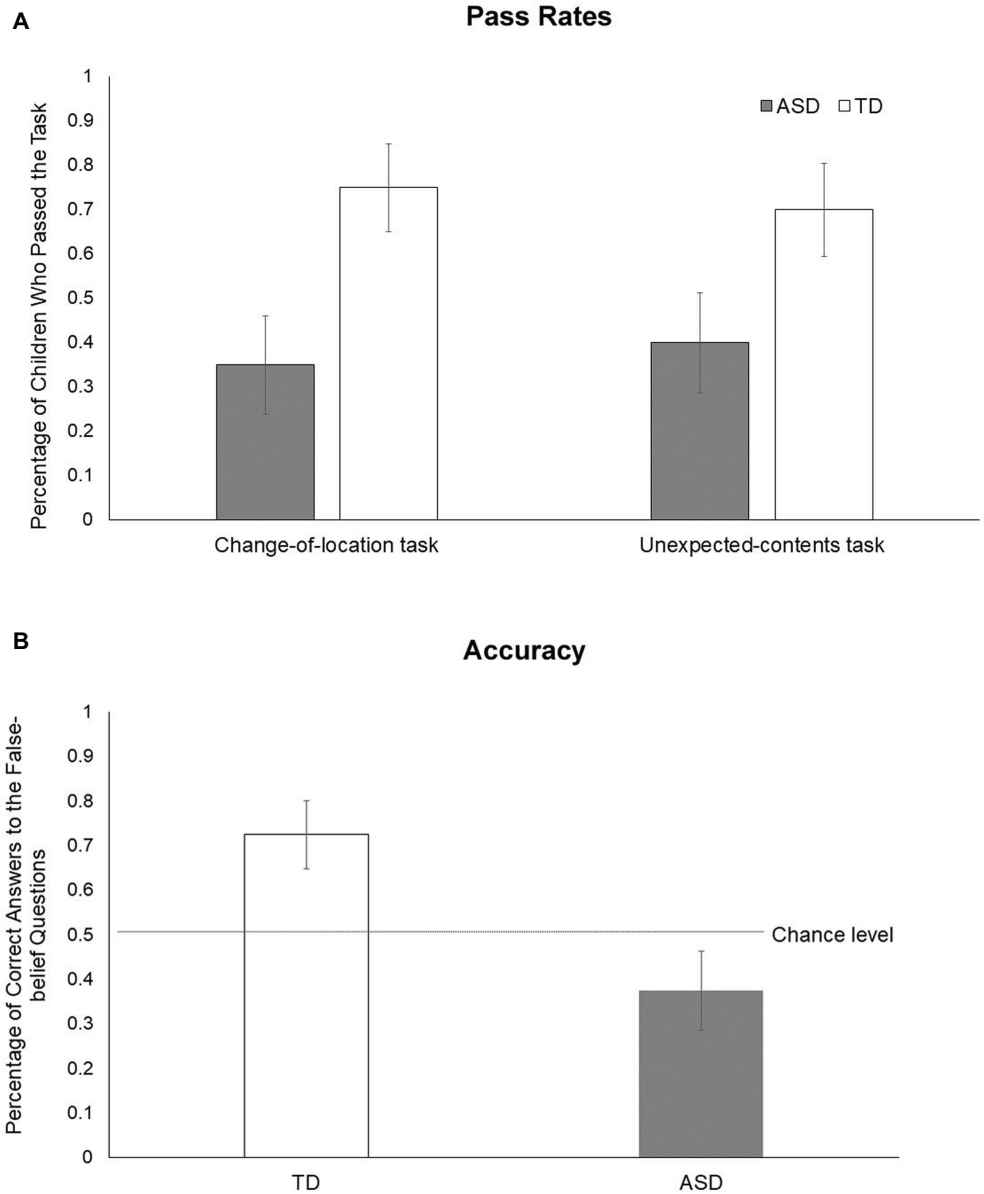
Pass rates **(A)** and accuracy **(B)** of the ASD and the TD groups.

Figure 2A shows the *pass rates* for each task: 45% of children with ASD failed in both tasks, and 20% passed both tasks; 10% of TD children failed to pass both tasks, and 55% of them succeeded in both tasks. We further conducted the Friedman test and found that the *pass rates* are similar between the two tasks, *p* = 1.0.

Figure 2B shows the *accuracy* for each group. We then compared the *accuracy* of each group to the chance level (i.e., 50%) and discovered that TD children’s *accuracy* (*M* = 0.73, SD = 0.34) was significantly higher than the chance level (0.5), *t*(19) = 2.93, *p =* 0.009; however, ASD children’s *accuracy* (*M* = 0.38, SD = 0.39) was similar to the chance level, *t*(19) = −1.42, *p =* 0.171. A Mann–Whitney *U* test found that the *accuracy* of the TD group (*M* = 0.73, SD = 0.34) was significantly higher than the ASD group (*M* = 0.38, SD = 0.39), *U* = 294.50, *W* = 504.50, *p =* 0.009. These findings suggest that like their deficits in interpreting other persons’ mental states in traditional false belief tasks, children with ASD are less likely than TD children to attribute mental states to the social robot.

For the categorization task, the percentages of children dividing the robot into toy, machine, human, and animal type were 50, 20, 20, and 10% for the ASD group, and 40, 40, 10, and 10% for the TD group. We conducted a chi-square test to examine the group difference in their categorization, and found that the two groups categorized the social robot similarly, *χ*^2^(3) = 2.26, *p* = 0.520. Using the Pearson correlation coefficients, we found no significant correlation between the children’s performance on the false belief tasks and categorization task, for ASD group: *r*(20) = 0.06, *p* = 0.817, and for TD group: *r*(20) = −0.30, *p* = 0.195.

## Discussion

This research tested whether children with and without ASD attributed false belief to the social robot in traditional false belief tasks. The results showed that most 5- to 7-year-old TD children could attribute false belief to the social robot. This understanding of the robot’s false belief is similar to the understanding of false belief of real persons or puppets representing persons in children of a similar age to those reported in previous literature (e.g., Wellman et al., 2001; Yi et al., 2014). Previous research has manifested that TD children perceived robots similarly to human beings in some fundamental ways, for example, in biological properties (Kahn et al., 2006) and as social beings (Kahn et al., 2012). Our study further illustrated that TD children could attribute higher level mental states to the robot by interpreting its false belief and predict its action accordingly, which extend our understanding of TD children’s perception and cognition on social robots.

On the other hand, children with ASD’s accuracy of the false belief tasks was statistically below chance and lower than that of the TD group, which confirmed that children with ASD still have difficulty in interpreting robots’ mental states and behaviors. This finding might derive from two possibilities. First, their impairments in ToM hindered the children with ASD from inferring the mental states of any agent, including the social robot. ToM deficits in children with ASD were believed to be correlated with their language development (e.g., discourse ability, Hale and Tager-Flusberg, 2005) or executive functions (Pellicano, 2007). An alternative possibility is that children with ASD perceived the robots differently from TD children did. Our results from the categorization task elucidated that both groups tended to classify the social robot into similar categories, which violates this alternative possibility. However, our findings seem to contradict the previous evidence of a different perception of robots in children with ASD and TD children (e.g., Peca et al., 2014). This difference in findings may derive from children’s first-hand experience with the robot: Peca et al. (2014) asked children to accomplish the categorization task after watching an introductive video about robots, while in our study, children finished the task after having social interactions with a robot in person. Therefore, we still could not rule out this alternative explanation and it still calls for future investigations to further test children’s anthropomorphic thinking of robots and its relation with their false belief understanding to better examine this possibility.

It should be noted that our study still has some limitations. First, we did not involve a human condition to compare with the robot condition investigated in this paper, though previous literature have shown that children with ASD were less likely to attribute false belief to another person in these two FB tasks (e.g., Jeffrey Farrar et al., 2017). Future studies that can directly compare children’s understanding of false belief of a robot to that of a person would provide more insights on how to effectively utilize the robot in potential intervention situations. The second limitation is on false belief task design. We investigated children’s performance, which involves interactions with the robot. On the other hand, the traditional false belief tasks were designed for the case, where children passively observe the puppet’s performance (e.g., Wimmer and Perner, 1983). Although interactive tasks made the experimental setting similar to the intervention situations, it still calls for future work that could simultaneously involve a passive observing task (e.g., two robots playing) and an interactive task (e.g., the participating child playing with the robot), leading to a more comprehensive analysis on children’s attribution to false belief of the robot. Third, the current findings were based on simple ToM tasks and did not allow us to probe the various factors impacting on children’s false belief understanding to robots. For example, executive function and language were reported to correlate with false belief performance of children with ASD (e.g., Hale and Tager-Flusberg, 2005; Pellicano, 2007). Future research could use these supplemental measures to examine the relationship between children’s socio-cognitive development to underlie the false belief understanding of ASDs. Last, the current false belief task design also has limitations to investigate why robots are particularly engaging to children with ASD and the etiological theories of ASD, e.g., Intense World Theory (Markram and Markram, 2010) or Social Motivation Theory of Autism (Chevallier et al., 2012). Future studies are thus recommended to address these important aspects.

In sum, by modifying two traditional false belief tasks, we found that children with ASD were less capable of understanding a robot’s false belief compared to TD children. Our study implied that although the social robot could greatly attract attention and interest of children with ASD (Dautenhahn and Billard, 2002; Scassellati et al., 2012), they may still have difficulty to infer the mental states when interacting with the social robot. This should be taken into considerations when designing a robot-based intervention approach to improve social behaviors of ASDs.

## Data Availability

The datasets generated for this study are available on request to the corresponding author.

## Ethics Statement

This study was carried out in accordance with the recommendations of “International Committee of Medical Journal Editors guidelines, Committee for Protecting Human and Animal Subjects, School of Psychological and Cognitive Sciences, Peking University” with written informed consent from all subjects. All subjects gave written informed consent in accordance with the Declaration of Helsinki. The protocol was approved by the “Committee for Protecting Human and Animal Subjects, School of Psychological and Cognitive Sciences, Peking University.”

## Author Contributions

YZ mainly collected and analyzed the data, drafted the manuscript. YZ, WS, ZT, SH, YW, and CL contributed to the data collection. QX proofread the manuscript. JC and LY designed the study and revised the manuscript.

### Conflict of Interest Statement

The authors declare that the research was conducted in the absence of any commercial or financial relationships that could be construed as a potential conflict of interest.

## Footnotes

^1^In Mandarin, “he” “she” and “it” are all pronounced as “ta1”, in which case children would not have a bias about the human-likeness of the robot from the introduction.
